# 4-(2-Methyl­anilino)pent-3-en-2-one

**DOI:** 10.1107/S1600536810021045

**Published:** 2010-06-05

**Authors:** Gertruida J. S. Venter, Gideon Steyl, Andreas Roodt

**Affiliations:** a Department of Chemistry, University of the Free State, PO Box 339, Bloemfontein 9300, South Africa

## Abstract

The title enamino ketone, C_12_H_15_NO, a derivative of 4-(phenyl­amino)­pent-3-en-2-one, presents a roughly planar [greatest displacement of an atom from the pentenone plane is 0.033 (2) Å] pentenone backbone, enhanced by an intra­molecular N—H⋯O hydrogen bond; the asymmetry in C—C distances in the group suggests the presence of unsaturated bonds. The overall geometry in the free ligand differs significantly from that in other reported compounds, in which it is coordinated to rhodium; this is reflected in the bond distances [the N⋯O distance is significantly increased (0.2 Å) upon coordination to the metal] and the dihedral angle between the benzene ring and the pentenone backbone [49.53 (5)°]. All of the methyl goups are rotationally disordered over two orientations of equal occupancy.

## Related literature

For synthetic background, see: Shaheen *et al.* (2006 ▶). For applications of enamino­ketones in liquid crystals, see: Pyżuk *et al.* (1993 ▶), in fluorescence, see: Xia *et al.* (2008 ▶), in complexes of medical inter­est, see: Tan *et al.* (2008 ▶); Chen & Rhodes (1996 ▶), in catalysis, see: Nair *et al.* (2002 ▶); Van Aswegen *et al.* (1991 ▶); Steyn *et al.* (1992 ▶, 1997 ▶); Otto *et al.* (1998 ▶); Roodt & Steyn (2000 ▶); Brink *et al.* (2010 ▶). For the structures of related ligand systems, see: Damoense *et al.* (1994 ▶); Venter *et al.* (2009*a* ▶,*b* ▶). 
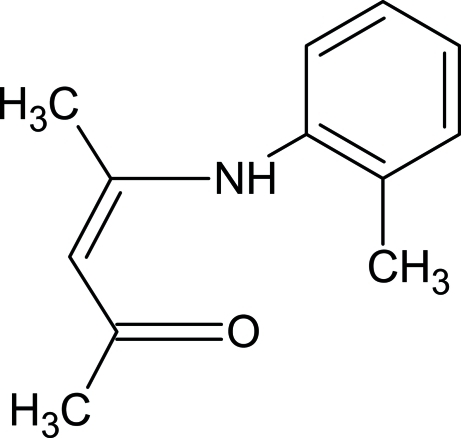


## Experimental

### 

#### Crystal data

C_12_H_15_NO
*M* *_r_* = 189.25
Monoclinic, 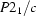

*a* = 7.5674 (7) Å
*b* = 11.5075 (9) Å
*c* = 12.0996 (11) Å
β = 92.154 (5)°
*V* = 1052.91 (16) Å^3^
*Z* = 4
Mo *K*α radiation
μ = 0.08 mm^−1^
*T* = 100 K
0.55 × 0.23 × 0.12 mm

#### Data collection

Bruker X8 APEXII 4K Kappa CCD diffractometer
Absorption correction: multi-scan (*SADABS*; Bruker, 2004 ▶) *T*_min_ = 0.960, *T*_max_ = 0.991
10022 measured reflections
2308 independent reflections
1854 reflections with *I* > 2σ(*I*)
*R*_int_ = 0.034

#### Refinement

*R*[*F* ^2^ > 2σ(*F* ^2^)] = 0.053
*wR*(*F* ^2^) = 0.150
*S* = 1.07
2308 reflections
129 parameters
H-atom parameters constrained
Δρ_max_ = 0.45 e Å^−3^
Δρ_min_ = −0.35 e Å^−3^

### 

Data collection: *APEX2* (Bruker, 2005 ▶); cell refinement: *SAINT-Plus* (Bruker, 2004 ▶); data reduction: *SAINT-Plus*; program(s) used to solve structure: *SHELXS97* (Sheldrick, 2008 ▶); program(s) used to refine structure: *SHELXL97* (Sheldrick, 2008 ▶); molecular graphics: *DIAMOND* (Brandenburg & Putz, 2005 ▶); software used to prepare material for publication: *WinGX* (Farrugia, 1999 ▶).

## Supplementary Material

Crystal structure: contains datablocks global, I. DOI: 10.1107/S1600536810021045/bg2348sup1.cif

Structure factors: contains datablocks I. DOI: 10.1107/S1600536810021045/bg2348Isup2.hkl

Additional supplementary materials:  crystallographic information; 3D view; checkCIF report

## Figures and Tables

**Table 1:** Hydrogen-bond geometry (Å, °)

*D*—H⋯*A*	*D*—H	H⋯*A*	*D*⋯*A*	*D*—H⋯*A*
N11—H11⋯O12	0.91	1.90	2.6345 (19)	136

**Table 2:** Comparative geometrical parameters (Å, °) for free and coordinated *N*,*O*-bidendate (*N*,*O*-bid) compounds

Parameters	(I)	(II)	(III)	(IV)
C111—N11	1.422 (2)	1.521 (4)/1.463 (3)	1.440 (4)	-
C2—N11	1.345 (2)	1.320 (4)	1.319 (4)	1.303 (6)
C4—O12	1.257 (2)	1.290 (3)	1.291 (4)	1.281 (6)
C2—C3	1.383 (3)	1.410 (4)	1.423 (4)	1.396 (7)
C3—C4	1.420 (2)	1.365 (3)	1.382 (3)	1.388 (9)
O12⋯N11	2.635 (2)	2.885 (3)	2.886 (3)	2.826 (6)
N11—C2—C4—O12	−0.5 (1)	4.1 (2)	−2.6 (2)	1.2 (4)
Dihedral angle	49.53 (5)	87.47 (4)/89.36 (8)	85.58 (8)	-

Notes: (I) This work; (II) *N*,*O*-bid = 4-(2,3-dimethyl phenyl­amino)­pent-3-en-2-onato (Venter *et al.*, 2009*a* ▶); (III) *N*,*O*-bid = 4-(2,6-dimethyl phenyl­amino)­pent-3-en-2-onato (Venter *et al.*, 2009*b* ▶). (IV) *N*,*O*-bid = 4-amino-pent-3-en-2-onato (Damoense *et al.*, 1994 ▶).   The dihedral angle is defined as the torsion angle between the N–C–C–C–O plane and the benzene ring. A positive angle denotes a clockwise rotation.

**Fig. 1.**
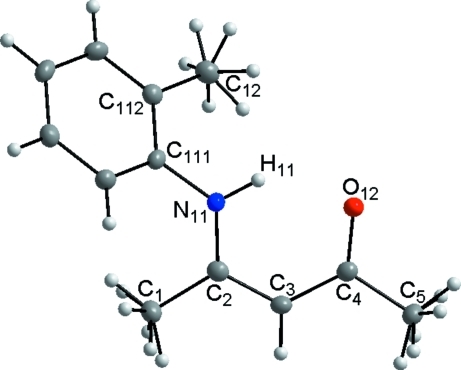
Molecular structure of the title compound. Displacement ellipsoids are drawn at the 50% probability displacement level.

## supplementary crystallographic information

### Comment

A well-known system in organometallic chemistry is the β-diketone compound AcacH (acetylacetone; or when coordinated acetylacetonato, acac^-^). A multitude of derivatives have been synthesized to date, with enaminoketones being one type. Since enaminoketones contain nitrogen and oxygen atoms as well as an unsaturated C—C bond, these electron-rich compounds are of interest in various fields including liquid crystals [Pyżuk *et al.* (1993)], fluorescence studies [Xia *et al.* (2008)] as well as formation of complexes of medical interest [Tan *et al.* (2008); Chen & Rhodes (1996)]. It also has significant application possibilities in catalysis [Nair *et al.* (2002); Van Aswegen *et al.* (1991); Steyn *et al.* (1992; 1997); Otto *et al.* (1998); Roodt & Steyn (2000); Brink *et al.* (2010)].

The title enaminoketone is a derivative of 4-(phenylamino)pent-3-en-2-one [PhonyH; Shaheen *et al.* (2006)]. Fig. 1 shows a view of the molecule. The C_2_–C_3_ distance of 1.383 (3) Å, versus the C_3_–C_4_ distance of 1.420 (2)Å indicates an unsaturated bond in the pentenone backbone, which is otherwise planar, probably helped by an intramolecular N_1_—H_1_···O_1_ bond (N_1_—H_1:_ 0.91Å; H_1_···O_1_: 1.90Å; N_1···_O_1_:2.635 (2)Å, N_1_—H_1_···O: 136.4°). In general terms, the geometry in the free ligand differs significantly from that in other reported compounds where it is coordinated to rhodium [Table1; Venter *et al.* (2009a; 2009b); Damoense *et al.* (1994)]; fon instance, the N_1_···O_1_ distance is greatly ncreased (~0.2 Å) upon coordination to the metal, as it is the dihedral angle between the phenyl ring and the pentenone backbone. All the methyl goups appear rotationally disordered in two sites of similar occupation.

### Experimental

A solution of acetylacetone (11.07 g, 0.1106 mol), 2-Me-aniline (10.73 g, 0.1008 mol) and 2 drops of H_2_SO_4_(conc.) in 150 ml benzene was refluxed for 6 hours in a DeanStark trap, filtered and left to crystallize. Crystals suitable for X-Ray diffraction were obtained in 17.86 g (94.32 %) yield. This compound is stable in air and light over a period of several months.

### Refinement

The methyl and aromatic H atoms were placed in geometrically idealized positions and constrained to ride on their parent atoms, with C—H = 0.95 and 0.98Å and U_iso_(H) = 1.5U_eq_(C) and 1.2U_eq_(C), respectively. The methyl groups were generated to fit the difference electron density and the groups were then refined as rigid rotors. The highest residual electron-density peak is 0.47Å from H1F.

### Crystal data

**Table:**

C_12_H_15_NO	*F*(000) = 408
*M**_r_* = 189.25	*D*_x_ = 1.194 Mg m^−^^3^
Monoclinic, *P*2_1_/*c*	Mo *K*α radiation, λ = 0.71073 Å
Hall symbol: -P 2ybc	Cell parameters from 2889 reflections
*a* = 7.5674 (7) Å	θ = 2.7–28.2°
*b* = 11.5075 (9) Å	µ = 0.08 mm^−^^1^
*c* = 12.0996 (11) Å	*T* = 100 K
β = 92.154 (5)°	Plate, colourless
*V* = 1052.91 (16) Å^3^	0.55 × 0.23 × 0.12 mm
*Z* = 4	

### Data collection

**Table:**

Bruker X8 APEXII 4K Kappa CCD diffractometer	2308 independent reflections
Radiation source: fine-focus sealed tube	1854 reflections with *I* > 2σ(*I*)
graphite	*R*_int_ = 0.034
ω and φ scans	θ_max_ = 27°, θ_min_ = 2.4°
Absorption correction: multi-scan (*SADABS*; Bruker, 2004)	*h* = −9→9
*T*_min_ = 0.960, *T*_max_ = 0.991	*k* = −14→12
10022 measured reflections	*l* = −15→15

### Refinement

**Table:**

Refinement on *F*^2^	Primary atom site location: structure-invariant direct methods
Least-squares matrix: full	Secondary atom site location: difference Fourier map
*R*[*F*^2^ > 2σ(*F*^2^)] = 0.053	Hydrogen site location: inferred from neighbouring sites
*wR*(*F*^2^) = 0.150	H-atom parameters constrained
*S* = 1.07	*w* = 1/[σ^2^(*F*_o_^2^) + (0.0654*P*)^2^ + 0.9506*P*] where *P* = (*F*_o_^2^ + 2*F*_c_^2^)/3
2308 reflections	(Δ/σ)_max_ < 0.001
129 parameters	Δρ_max_ = 0.45 e Å^−^^3^
0 restraints	Δρ_min_ = −0.35 e Å^−^^3^

### Special details

**Table:**

Experimental. The intensity data was collected on a Bruker X8 ApexII 4 K Kappa CCD diffractometer using an exposure time of 60 seconds/frame. A total of 688 frames were collected with a frame width of 0.5° covering up to θ = 28.24° with 99.1% completeness accomplished.
Geometry. All e.s.d.'s (except the e.s.d. in the dihedral angle between two l.s. planes) are estimated using the full covariance matrix. The cell e.s.d.'s are taken into account individually in the estimation of e.s.d.'s in distances, angles and torsion angles; correlations between e.s.d.'s in cell parameters are only used when they are defined by crystal symmetry. An approximate (isotropic) treatment of cell e.s.d.'s is used for estimating e.s.d.'s involving l.s. planes.

### Fractional atomic coordinates and isotropic or equivalent isotropic displacement parameters (Å^2^)

**Table:**

	*x*	*y*	*z*	*U*_iso_*/*U*_eq_	Occ. (<1)
C1	0.4288 (3)	0.30725 (17)	0.73241 (16)	0.0212 (4)	
H1A	0.4068	0.3907	0.723	0.032*	0.5
H1B	0.3886	0.2659	0.6653	0.032*	0.5
H1C	0.5557	0.2939	0.7458	0.032*	0.5
H1D	0.4939	0.243	0.6998	0.032*	0.5
H1E	0.5121	0.3678	0.7575	0.032*	0.5
H1F	0.345	0.3397	0.6769	0.032*	0.5
C2	0.3300 (2)	0.26340 (16)	0.82877 (15)	0.0172 (4)	
C3	0.3410 (2)	0.14730 (15)	0.85770 (15)	0.0174 (4)	
H3	0.4142	0.0982	0.8159	0.021*	
C4	0.2494 (2)	0.09702 (15)	0.94616 (14)	0.0162 (4)	
C5	0.2647 (3)	−0.03183 (16)	0.96633 (16)	0.0217 (4)	
H5A	0.1945	−0.0532	1.0296	0.033*	0.5
H5B	0.3889	−0.0521	0.982	0.033*	0.5
H5C	0.2208	−0.0739	0.9005	0.033*	0.5
H5D	0.3417	−0.0663	0.9118	0.033*	0.5
H5E	0.1472	−0.0674	0.9594	0.033*	0.5
H5F	0.3153	−0.0456	1.0409	0.033*	0.5
C12	0.2730 (3)	0.49042 (17)	1.07167 (15)	0.0218 (4)	
H12A	0.2961	0.4067	1.0685	0.033*	0.5
H12B	0.1797	0.5055	1.1238	0.033*	0.5
H12C	0.3812	0.5311	1.0964	0.033*	0.5
H12D	0.2752	0.5555	1.1239	0.033*	0.5
H12E	0.3917	0.4567	1.0687	0.033*	0.5
H12F	0.1901	0.4311	1.0961	0.033*	0.5
O12	0.15430 (17)	0.15514 (11)	1.00878 (10)	0.0195 (3)	
C111	0.1994 (2)	0.45704 (15)	0.86788 (15)	0.0160 (4)	
C112	0.2147 (2)	0.53330 (16)	0.95849 (15)	0.0172 (4)	
C113	0.1737 (2)	0.64979 (16)	0.94050 (16)	0.0203 (4)	
H113	0.1821	0.7026	1.0008	0.024*	
C114	0.1207 (2)	0.69078 (16)	0.83647 (16)	0.0208 (4)	
H114	0.0932	0.7707	0.8262	0.025*	
C115	0.1081 (2)	0.61469 (16)	0.74784 (15)	0.0192 (4)	
H115	0.0738	0.6426	0.6763	0.023*	
C116	0.1456 (2)	0.49751 (16)	0.76367 (15)	0.0180 (4)	
H116	0.1344	0.445	0.7032	0.022*	
N11	0.2320 (2)	0.33691 (13)	0.88747 (13)	0.0176 (3)	
H11	0.1801	0.3060	0.9471	0.021*	

### Atomic displacement parameters (Å^2^)

**Table:**

	*U*^11^	*U*^22^	*U*^33^	*U*^12^	*U*^13^	*U*^23^
C1	0.0225 (9)	0.0206 (9)	0.0210 (9)	0.0019 (7)	0.0057 (7)	0.0032 (7)
C2	0.0167 (8)	0.0190 (9)	0.0159 (9)	0.0001 (7)	0.0001 (7)	−0.0007 (7)
C3	0.0192 (9)	0.0163 (9)	0.0168 (9)	0.0028 (7)	0.0029 (7)	−0.0015 (7)
C4	0.0176 (8)	0.0157 (9)	0.0152 (8)	0.0008 (7)	−0.0013 (7)	−0.0010 (7)
C5	0.0254 (10)	0.0152 (9)	0.0250 (10)	0.0014 (7)	0.0062 (8)	0.0005 (7)
C12	0.0221 (9)	0.0244 (10)	0.0188 (9)	−0.0010 (8)	0.0000 (7)	−0.0015 (7)
O12	0.0254 (7)	0.0157 (6)	0.0176 (7)	0.0015 (5)	0.0051 (5)	−0.0001 (5)
C111	0.0158 (8)	0.0122 (8)	0.0202 (9)	−0.0008 (6)	0.0032 (7)	0.0003 (7)
C112	0.0164 (8)	0.0177 (9)	0.0177 (9)	−0.0018 (7)	0.0023 (7)	−0.0002 (7)
C113	0.0222 (9)	0.0170 (9)	0.0220 (9)	−0.0018 (7)	0.0033 (7)	−0.0059 (7)
C114	0.0214 (9)	0.0129 (8)	0.0284 (10)	0.0004 (7)	0.0047 (8)	0.0012 (7)
C115	0.0189 (9)	0.0190 (9)	0.0198 (9)	−0.0001 (7)	0.0024 (7)	0.0031 (7)
C116	0.0200 (9)	0.0163 (9)	0.0178 (9)	−0.0020 (7)	0.0020 (7)	−0.0018 (7)
N11	0.0232 (8)	0.0132 (7)	0.0168 (8)	0.0009 (6)	0.0053 (6)	0.0012 (6)

### Geometric parameters (Å, °)

**Table:**

C1—C2	1.496 (2)	C12—C112	1.506 (3)
C1—H1A	0.98	C12—H12A	0.98
C1—H1B	0.98	C12—H12B	0.98
C1—H1C	0.98	C12—H12C	0.98
C1—H1D	0.98	C12—H12D	0.98
C1—H1E	0.98	C12—H12E	0.98
C1—H1F	0.98	C12—H12F	0.98
C2—N11	1.345 (2)	C111—C116	1.391 (3)
C2—C3	1.383 (3)	C111—C112	1.406 (3)
C3—C4	1.420 (2)	C111—N11	1.422 (2)
C3—H3	0.95	C112—C113	1.391 (3)
C4—O12	1.257 (2)	C113—C114	1.389 (3)
C4—C5	1.506 (2)	C113—H113	0.95
C5—H5A	0.98	C114—C115	1.385 (3)
C5—H5B	0.98	C114—H114	0.95
C5—H5C	0.98	C115—C116	1.390 (3)
C5—H5D	0.98	C115—H115	0.95
C5—H5E	0.98	C116—H116	0.95
C5—H5F	0.98	N11—H11	0.9071
			
C2—C1—H1A	109.5	H5B—C5—H5F	56.3
C2—C1—H1B	109.5	H5C—C5—H5F	141.1
H1A—C1—H1B	109.5	H5D—C5—H5F	109.5
C2—C1—H1C	109.5	H5E—C5—H5F	109.5
H1A—C1—H1C	109.5	C112—C12—H12A	109.5
H1B—C1—H1C	109.5	C112—C12—H12B	109.5
C2—C1—H1D	109.5	H12A—C12—H12B	109.5
H1A—C1—H1D	141.1	C112—C12—H12C	109.5
H1B—C1—H1D	56.3	H12A—C12—H12C	109.5
H1C—C1—H1D	56.3	H12B—C12—H12C	109.5
C2—C1—H1E	109.5	C112—C12—H12D	109.5
H1A—C1—H1E	56.3	H12A—C12—H12D	141.1
H1B—C1—H1E	141.1	H12B—C12—H12D	56.3
H1C—C1—H1E	56.3	H12C—C12—H12D	56.3
H1D—C1—H1E	109.5	C112—C12—H12E	109.5
C2—C1—H1F	109.5	H12A—C12—H12E	56.3
H1A—C1—H1F	56.3	H12B—C12—H12E	141.1
H1B—C1—H1F	56.3	H12C—C12—H12E	56.3
H1C—C1—H1F	141.1	H12D—C12—H12E	109.5
H1D—C1—H1F	109.5	C112—C12—H12F	109.5
H1E—C1—H1F	109.5	H12A—C12—H12F	56.3
N11—C2—C3	120.21 (16)	H12B—C12—H12F	56.3
N11—C2—C1	120.06 (16)	H12C—C12—H12F	141.1
C3—C2—C1	119.73 (16)	H12D—C12—H12F	109.5
C2—C3—C4	123.91 (16)	H12E—C12—H12F	109.5
C2—C3—H3	118	C116—C111—C112	120.72 (16)
C4—C3—H3	118	C116—C111—N11	121.30 (16)
O12—C4—C3	122.97 (16)	C112—C111—N11	117.91 (16)
O12—C4—C5	117.93 (16)	C113—C112—C111	117.91 (17)
C3—C4—C5	119.10 (15)	C113—C112—C12	120.93 (16)
C4—C5—H5A	109.5	C111—C112—C12	121.16 (16)
C4—C5—H5B	109.5	C114—C113—C112	121.61 (17)
H5A—C5—H5B	109.5	C114—C113—H113	119.2
C4—C5—H5C	109.5	C112—C113—H113	119.2
H5A—C5—H5C	109.5	C115—C114—C113	119.74 (17)
H5B—C5—H5C	109.5	C115—C114—H114	120.1
C4—C5—H5D	109.5	C113—C114—H114	120.1
H5A—C5—H5D	141.1	C114—C115—C116	119.93 (17)
H5B—C5—H5D	56.3	C114—C115—H115	120
H5C—C5—H5D	56.3	C116—C115—H115	120
C4—C5—H5E	109.5	C115—C116—C111	120.08 (17)
H5A—C5—H5E	56.3	C115—C116—H116	120
H5B—C5—H5E	141.1	C111—C116—H116	120
H5C—C5—H5E	56.3	C2—N11—C111	128.22 (16)
H5D—C5—H5E	109.5	C2—N11—H11	115.9
C4—C5—H5F	109.5	C111—N11—H11	115.9
H5A—C5—H5F	56.3		
			
N11—C2—C3—C4	1.9 (3)	C112—C113—C114—C115	−0.1 (3)
C1—C2—C3—C4	−178.62 (17)	C113—C114—C115—C116	1.1 (3)
C2—C3—C4—O12	−2.6 (3)	C114—C115—C116—C111	−1.4 (3)
C2—C3—C4—C5	177.01 (17)	C112—C111—C116—C115	0.7 (3)
C116—C111—C112—C113	0.3 (3)	N11—C111—C116—C115	177.51 (16)
N11—C111—C112—C113	−176.63 (16)	C3—C2—N11—C111	−177.32 (17)
C116—C111—C112—C12	−179.96 (16)	C1—C2—N11—C111	3.2 (3)
N11—C111—C112—C12	3.1 (3)	C116—C111—N11—C2	48.9 (3)
C111—C112—C113—C114	−0.6 (3)	C112—C111—N11—C2	−134.19 (19)
C12—C112—C113—C114	179.64 (17)		

### Hydrogen-bond geometry (Å, °)

**Table:**

*D*—H···*A*	*D*—H	H···*A*	*D*···*A*	*D*—H···*A*
N11—H11···O12	0.91	1.90	2.6345 (19)	136

### Table 2 Comparative geometrical parameters (Å, °) for free and coordinated *N*,*O*-bidendate (*N*,*O*-bid) compounds

**Table:**

Parameters	(I)	(II)	(III)	(IV)
N11—C111	1.422 (2)	1.521 (4)/1.463 (3)	1.440 (4)	-
N11—C2	1.345 (2)	1.320 (4)	1.319 (4)	1.303 (6)
O12—C4	1.257 (2)	1.290 (3)	1.291 (4)	1.281 (6)
C2—C3	1.383 (3)	1.410 (4)	1.423 (4)	1.396 (7)
C3—C4	1.420 (2)	1.365 (3)	1.382 (3)	1.388 (9)
N11···O12	2.635 (2)	2.885 (3)	2.886 (3)	2.826 (6)
N11—C2—C4—O12	-0.5 (1)	4.1 (2)	-2.6 (2)	1.2 (4)
Dihedral angle	49.53 (5)	87.47 (4)/89.36 (8)	85.58 (8)	-

Notes: (I) This work; (II) *N*,*O*-bid = 4-(2,3-dimethyl phenylamino)pent-3-en-2-onato (Venter *et al.*, 2009*a*); (III) *N*,*O*-bid = 4-(2,6-dimethyl phenylamino)pent-3-en-2-onato (Venter *et al.*, 2009*b*). (IV) *N*,*O*-bid = 4-amino-pent-3-en-2-onato (Damoense, *et al.*, 1994).
